# “You get three different hats on and try to figure it out:” home based care provision during a disaster

**DOI:** 10.1186/s12912-021-00676-2

**Published:** 2021-08-31

**Authors:** Sue Anne Bell, Sarah Dickey, Marie-Anne Rosemberg

**Affiliations:** University of Michigan School of Nursing, 400 North Ingalls Building, Ann Arbor, MI 48109 USA

**Keywords:** Home based care, Aging, Disaster, Health care quality

## Abstract

**Background:**

Home based care is a vital, and growing, part of the health care system that allows individuals to remain in their homes while still receiving health care. During a disaster, when normal health care systems are disrupted, home based care remains a vital source of support for older adults. The purpose of this paper is to qualitatively understand the barriers and facilitators of both patients and providers that influence the provision of home based care activities in two hurricane affected communities.

**Methods:**

Using qualitative inquiry informed by the social ecological model, five focus groups were conducted with home based care providers (*n* = 25) in two settings affected by Hurricane Irma and Hurricane Harvey. An open-source database of home health agencies participating in Centers for Medicare and Medicaid Services programs was used to identify participants. Data were manually coded and larger themes were generated from recurring ideas and concepts using an abductive analysis approach.

**Results:**

Twenty five participants were included in one of five focus groups. Of the 22 who responded to the demographic survey, 65 % were registered nurses, 20 % were Licensed Vocational Nurses (LVN), and 15 % were other types of health care providers. 12 % of the sample was male and 88 % was female. Five themes were identified in the analysis: *barriers to implementing preparedness plans*, *adaptability of home based care providers, disasters exacerbate inequalities*, *perceived unreliability of government and corporations*, and *the balance between caring for self and family and caring for patients.*

**Conclusions:**

This study provides qualitative evidence on the factors that influence home based care provision in disaster-affected communities, including the barriers and facilitators faced by both patients and providers in preparing for, responding to and recovering from a disaster. While home based care providers faced multiple challenges to providing care during and after a disaster, the importance of community supports and holistic models of care in the immediate period after the disaster were emphasized. We recommend greater inclusion of home health agencies in the community planning process. This study informs the growing body of evidence on the value of home based care in promoting safety and well-being for older adults during a disaster.

## Background

The United States population is rapidly aging. Currently, more than 46 million Americans are aged 65 years of age and older [1]. According to Healthy People 2020, this number is expected to reach 98 million by 2060 [2]. Aging comes along with its own set of unique challenges including an increase in chronic conditions such as diabetes, heart disease, and dementia, along with increased difficulty carrying out activities of daily living (ADLs). Given the unique needs that come with aging and the projected increase in numbers of older adults, it is imperative that older adults receive support that allows them to age optimally in place.

Healthy aging needs are compounded by the effects of disasters, such as hurricanes, where normal patterns of daily living are disrupted. Hurricane Harvey and Irma were two large-scale disasters critically affecting the coastal South of the United States in 2017. Responsible for an estimated 90 deaths and nearly $200 billion in damages [3], Hurricane Harvey is one of the most significant disasters of this century. During Hurricane Harvey, over 300,000 customers lost power lasting for some up to 2 weeks, and 20 hospitals closed temporarily [4, 3]. Hurricane Irma was similarly devastating, causing 6 million residents in Florida to be evacuated from coastal areas, and thousands of homes damaged [5]. These events not only have historic significance in terms of the amount damage and destruction, but they also have individual effects on older adults, their caregivers, and their communities.

Alongside an increasing number of large-scale disasters, and with the exponential increase in aging populations, the spectrum of home based care services is expected to grow as well [6]. Since home based care in particular is one area where emergency preparedness and response interventions can have substantial effects, expanded training for this workforce is important. Research efforts to improve disaster preparedness in home based care programs at the Veteran’s Health Administration have benefited from being part of a larger parent organization set up to provide longitudinal interdisciplinary care, where a strength is the implementation of early preparedness programming [7–11]. The majority—over 80 %— of home health agencies are operated as for-profit, where they may be only peripherally attached to an existing healthcare organization, often leaving emergency planning to the agency itself [12]. Regardless, health care at home is intended to continue to function during a disaster or community emergency, and home health care agencies receiving Medicare and Medicaid funding are mandated to have emergency preparedness plans in place [13]. During a disaster, home health care needs of patients continue, and home based care providers (HCPs) use existing care delivery models to support clients wherever they may be located, including in shelters and hotels. Every disaster presents unique challenges, and the unpredictable nature of disasters requires HCPs to adapt rapidly to changing situations in order to provide minimal interruptions in care. HCPs provide a vital lifeline for clients when their access to other types of healthcare may be restricted. Despite the importance of home based care, we know little about the experiences of home based care providers during a disaster, including the successful workarounds that are employed as well as the challenges to supporting clients that remain to be addressed. Therefore, the purpose of this paper is to qualitatively understand the barriers and facilitators of both patients and providers that influence the provision of home based care activities in two hurricane affected communities.

## Methods

This descriptive, qualitative study was part of a larger study that explored home based care providers’ experiences providing care during a disaster, and was informed by the social-ecological model [14, 15]. Institutional review board (IRB) approval was received from the University of Michigan (HUM00132531). Participants provided written informed consent and were offered a $50 visa gift card via email as an incentive for participation. This study adhered to the consolidated criteria for reporting qualitative research (COREQ) guidelines.

### Interview guide

An interview guide informed by prior conceptual work on disasters, home based care, and aging was developed [16–20]. This guide was refined through pilot testing with qualitative experts initially and then with a small group of registered nurses. The interview guide focused on barriers clients faced to health and healthcare access after the disaster, strategies to support preparedness before the disaster, and strategies after the disaster to support safety and aging in place. The final interview guide was constructed for a goal interview length of 45 to 60 min.

### Study sample and recruitment

The study sample was recruited from counties with Federal Emergency Management Agency (FEMA) disaster declarations for Hurricane Irma and Harvey [21], and further limited to counties that received individual assistance funding. Individual assistance provides support for individuals (rather than only for public assistance such as rebuilding roads) and was conceptualized in this study as a measure of the extent to which a disaster affected the community. We also considered county demographic statistics on socioeconomic status and race in our recruitment strategy. In order to include the perspective of the home based care workforce on caring for historically underserved patient populations, counties with higher numbers of residents who identify as Black, Hispanic, Asian, and “other” were prioritized in our recruitment process, as were counties with a median annual income closest to 2017 Federal poverty guidelines. In order to reach saturation, a sample size of 25 was targeted a priori.

An open-source database of home health agencies participating in Centers for Medicare and Medicaid Services (CMS) programs was used to identify and recruit participants. A two-stage sampling design was employed. First, we contacted the 164 home health agencies that met the study criteria. Each agency was contacted via telephone by a member of the study team up to five times. After a fifth unanswered call or request for a call back, the agency was removed from the study due to non-response. Once contact was made, the study was explained to the agency administrator or their representative, and study information was disseminated to potential participants, who then contacted the study team. From these phone calls, twenty-five home based care providers were recruited to attend one of five focus groups.

### Focus groups

Five focus groups were conducted in person in January, October, and November 2019 in greater Houston, Texas, and in Southern Florida. The focus group participants had provided care during hurricanes Harvey and Irma, both of which made landfall in the late summer of 2017. Each focus group was moderated by the principal investigator who has doctoral-level training in qualitative methods, with support from a trained research assistant, who was responsible for the audio recording, informed consent and incentive documents, and note-taking. Focus groups lasted approximately 60 min and started with an introduction, during which participants were encouraged to speak openly, written informed consent was obtained, and then followed by the semi-structured interview itself.

### Analysis

**Fig. 1:**
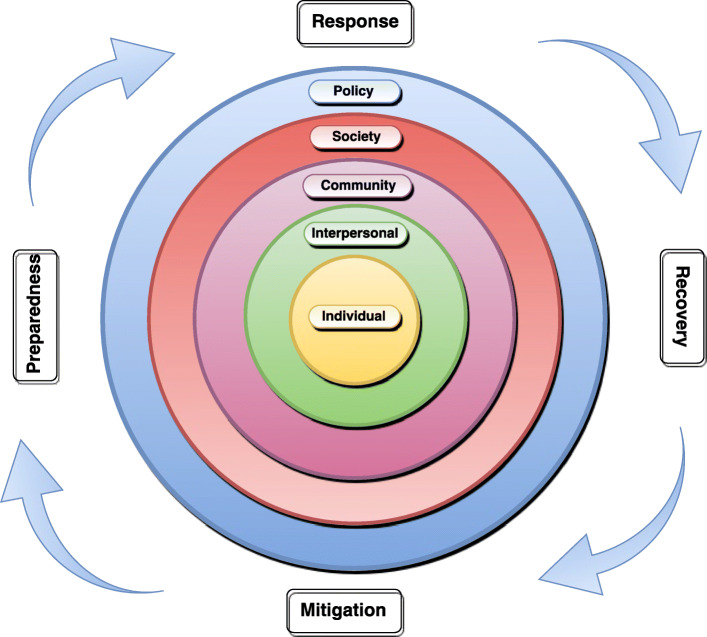
Theoretical framework

After the study team reached an agreement that data from the focus groups had reached saturation, the processes of coding and analysis were initiated. Focus group conversations were recorded digitally and transcribed by an IRB-approved transcription service. After removing identifying information from the transcripts, including the mention of a specific facility, other clinicians, or family members, the transcripts were formatted for coding. Data was analyzed using an abductive analytic approach [22]. This method combines inductive and deductive approaches thereby allowing for a purposeful examination of a range of explanations, which reduces the likelihood of bias. Using an iterative process, two research assistants independently generated codes in light of existing theory, here using the social-ecological model [23] applied to the disaster life cycle of mitigation, preparedness, response, and recovery (Fig. 1). Coders met face-to-face weekly in order to review and arbitrate differences in each other’s codes. The final codes and their agreed upon definitions were then entered into a codebook. These codes were presented to the larger research team, and after systematic analysis and collective deliberation, five larger themes emerged from the data that represented larger over-arching concepts extracted from the focus groups transcript data. These themes represent common strategies used by participants and the barriers they faced in providing care for older adults during disasters.

## Results

### Demographics

The study sample was drawn from an open database of home health agencies participating in CMS programs, and as such all participants were employees of CMS-certified home health agencies. A total of 25 participants were included across five focus groups, with five home health agencies represented. Of the 22 who responded to the demographic survey, 12 % were male and 88 % were female. The majority (84 %) identified as White/Caucasian, followed by 12 % who identified as Hispanic, and 4 % as Black. Registered nurses made up 65 % of the sample, 20 % were Licensed Vocational Nurses (LVN) and 15 % were other healthcare roles, including physical therapy and administration. The average length of time participants resided in their profession at the time of the focus group was 16.5 years, with a range of 3 to 39 years. Three participants did not complete the demographic survey.

### Themes

Five themes were generated from the analysis. These themes represent the experiences of home based care providers from CMS-certified home health agencies during two hurricane disasters, and addresses the barriers they faced in providing care for older adults during these events and the successful strategies they employed.

#### Barriers to implementing preparedness plans

Participants described the extensive investment in preparedness planning with their patients, with agreement across participants. They described preparedness plans which were communicated to clients upon admission into home health services. These tailored plans are designed specifically for a client’s care needs and also provide information on community resources and education for them. Participants described preparedness planning:

“We do all this on admission. We get the emergency plan put in place for each patient. It’s individualized. Everything’s on that plan. Then, we also give them emergency management packages which is information packages. Everything from hurricane tracking information, all the way through to what to have as emergency storage, foods, things like that. All in this little booklet that we give them. It comes in multiple different languages.” (C3).

“We confirm what their evacuation plan is, and talk with them about, okay, you have to leave, where you going to go? What are you going to bring with you? How are you going to get there? What are you going to do?” (B3).

However, despite the efforts of participants to prepare their clients, barriers existed around supporting clients in implementing the plans, and notably, across all programs, participants shared these challenges. Participants cited reasons such as financial concerns, lack of transportation, and health and mobility issues, but also held the view that many believed the storm would not cause substantial damage.

“And so those types of preparations, I don’t know that we could do any better just because of the way the world works. You tell people to have 2 weeks extra of their medicines, but there’s really no way to do that. That’s a real problem.” (B4).

“Then, like I said, you’ll get the call from the family. It’s like, ‘We just don’t know what to do. ‘Really? We’ve been at you for a week… giving you information. We’ve prepared you since admission.’”(C3).

#### Adaptability of home based care providers

In describing their disaster response actions, HCPs reported numerous instances of supporting client needs outside of healthcare. Whether it was helping with disaster aid applications or navigating insurance websites on the internet, clients often had no other options for assistance with these tasks. HCPs stated they viewed these types of support for their clients as out of their expected role, but also a necessary part of keeping their clients healthy and in their homes.

“So then when you see the patients, you’re there to deal with their medical concerns and we’re supposed to be doing our wound care, whatever but you’re also having to deal with all the other stuff because that’s part of what they’re dealing with right now so then that gets thrown on you and so then you’re dealing with that right now because you don’t want to just leave them there with no type of solution or help. So then you get three different hats on and try to figure out how to take care of them.” (B5).

“She (the HCP) brought him diapers because she wasn’t sure whether they took them with them, when they evacuated. So she just brought diapers with her, just in case. She stopped off at a grocery store that was open and bought them herself. I mean, so, everybody… It was everything from just whoever was looking after the patient, jumped in. (C3)

#### Balance between caring for self and family and caring for patients

HCPs described experiencing multiple stressors as they juggled patients’ disaster-related needs with those that they and their own families were experiencing. Participants cited challenges with ensuring their patients’ well-being as well as that of their own families. Participants reported the critical importance of having personal preparedness plans in place for their own families to make sure they were taken care of while the provider was working with patients. They reported feelings of helplessness because they were not able to help patients regain lost possessions or provide answers while simultaneously caring for the same issues for themselves and their own families.

“So we’re calling all of our staff because one of the first things that we’ve got to make sure is that our staff is prepared in getting their family taking care of… So that they don’t have to worry about them, while they’re taking care of our patients.” (C2).

“You need to understand your own personal plan and you need to understand who’s responsible for your family while we need you here, so we have designated who’s at the beginning of the disaster, who comes back at the end of the disaster and how that works.” (B3).

“I was considerably more concerned about my patients than I was my family. I knew my family was basically okay, but my patients were not. My son lost everything in Hurricane Harvey but I knew that physically he was okay, and I knew that we would be able to help him and he was going to be okay. So for that reason I didn’t worry as much about my family, but my patients, I can’t fix those kinds of problems for them.” (B2).

#### Disasters exacerbate inequalities

Participants described how the disaster disrupted the way their clients access essential services and information, where such disruptions were described to have a greater impact on those living with disabilities, limited literacy, and/or low socioeconomic status. Many participants reported having clients who stayed home, were reluctant to evacuate, or lacked an acceptable place to evacuate to, citing mobility issues or poor experiences in previous evacuations. Participants described the difficulties in terms of both accessing essential services and information, as well as decision-making on taking action to use resources.

“The other thing I wanted to say is our elderly population here, a large percent do not write. They don’t write. They don’t know how to write because of their education level. Or read. So the FEMA program has to have people that can understand that to help these people.” (E4).

“What am I going to do? Where am I going to stay? I can’t afford a hotel, I don’t have any family anywhere else. This is where all my family is at. And so what they typically find is they find the family member who they think has the most sound structure. And that’s typically what they’ll do to ride out the storm and they’ll suffer through heat and humidity and mold if they have to, just to make sure that everybody’s safe.“ (E2).

Participants also stated that limited education around health and safety in the aftermath of disasters was also an issue. They described how this lack of education contributed to unsafe living conditions.

“If you have floodwaters in your house, you can’t be walking around with your bandaged foot, and there’s so many people doing that. So many people. Well first of all they didn’t have tools but also just not aware of the danger of doing that.” (B5).

#### Perceived unreliability of government and corporations

Participants expressed a consensus that federal resources (such as FEMA) and insurance companies were viewed with mistrust by their clients. A primary barrier to clients receiving disaster support was the application process, which they described as extraordinarily challenging, and even once completed, there was no follow-through about the status of the application. They also described confusion among their clients about next steps in their insurance claims or disaster applications, and poor communication in the event they were able to speak in-person to representatives.

“I think the other thing about that is that the application process was very complicated. We tried to pull a lot of community information for people because the County would publish, go here for FEMA help, go here to access. You’ve got to fill out these forms, you got to talk to these people. But it’s a very complicated process.” (B6).

“Many people told me the FEMA people got mad at them, which with some of our elderly people, you get mad at them, they’re done, they shut down, they’re done. They’re not going to be disrespectful. They’re just done. And so that’s an issue in my opinion.” (E4).

“And yet they paid in to their insurances all this year, never missed a payment, and are not getting any help either on that end.” (E6).

## Discussion

This study provides qualitative evidence on the factors that influence home based care provision in disaster-affected communities, including the barriers and facilitators faced by both patients and providers in preparing for, responding to, and recovering from a disaster. HCPs themselves are a part of the community of individuals affected, as they live in the disaster-affected communities where they work.

Home health agencies participating in Centers for Medicare and Medicaid programs, such as those in this study, are required to have disaster preparedness plans for the agency as a whole, and individual plans for patients. Components of these disaster preparedness plans may include evacuation planning, triage, storm and flood preparation planning, wound care education, resources and literature, alternative dialysis sites, specialized diet education, and communication with the home health care agency at the time of a disaster. Despite this careful planning, barriers remained, particularly around financial concerns, transportation, and health and mobility issues, but also based on attitudes and opinions formed by past disaster experiences.

This study also observed barriers around formal supports by their patients, particularly among interactions with insurance agencies and governmental resources. The associated complexities and delays seen with these formal supports were a source of frustration, and even despair, for the patient population served by HCPs. Notably, reducing complexity was a major stated goal in FEMA’s 2018 strategic plan [24]. Progress on actions to reduce complexity at the community level will benefit from both continuous evaluation and ongoing community stakeholder involvement. Mistrust of the government is not a new challenge, but is a place for still-needed intervention [25, 26]. This study can inform future policy advocating for closer cooperation with organizations that provide formal support, particularly around leveraging the trust of HCPs.

While our study guide was focused on how providers supported patients through the hurricanes, with questions around efforts to prevent chronic disease breakdowns and thereby avoid hospitalization, larger themes emerged about how to holistically support patients. Participants emphasized the value of the community itself, their own place as members of the community, historic mistrust in governmental agencies, and the challenges senior Americans face living on a fixed income. Using our abductive analytic approach [22]—which seeks to uncover surprising or anomalous findings—we were surprised to hear less about specific patient care experiences and the logistics around deploying preparedness plans (which did exist and were used), but instead more about the determination of providers to care for their patients in a resource-constrained setting, and about the barriers of fixed incomes, limited literacy, and distrust in formal response and recovery methods— in setting the course for recovery.

In keeping with our use of the social-ecological model alongside the disaster management cycle of mitigation, preparedness, response, and recovery, a significant effort at the policy level is needed to improve trust in order to better serve populations affected by disasters. Formal support agencies can focus on building relationships with local community leaders as a mitigation strategy. This could include strategic efforts with stakeholders from these organizations and community leaders on how to best support members of the community, with attention on how to improve disaster response and recovery service delivery at the local level. To further capitalize on local resource networks, formal support agencies can focus on how to work alongside community organizations, including having these local organizations take the lead on response and recovery, perhaps with financial support from formal agencies. Immediate action steps to take would be to include home health agencies into community-wide planning, training, and drills. This would perhaps have multiplicative positive impacts, including further preparing home based care providers and home health agencies, multiple groups learning from each other, and the establishment of formal and informal relationships that may arise from interacting together.

We also saw the importance, and value, of the relationship between the HCP, the client, and the community in this study. HCPs often provided support and assistance to clients outside of expected clinical practice, including help with filling out forms or navigating resources on the internet. HCPs had to adapt their practice after the disaster to address concerns outside of health, doing so with the knowledge that without this assistance, clients would be at risk for social issues that would ultimately affect their health. Given that HCPs often have established and trusting relationships with their clients, formal organizations could coordinate with home health agencies to understand the needs of their clients and develop shared strategies to support clients in promoting response and recovery.

Our study highlights the need for an ongoing focus on equitable solutions to support structurally marginalized communities throughout the disaster management cycle. Despite existing efforts by organizations targeted towards these communities and preparedness planning done by HCPs, our study found that many still reported experiencing inequities as a result of the disaster. Participants identified a need for strategies to support low-literacy populations. An area also identified as a need is for focused interventions around evacuation education for those who cannot or will not evacuate. Many participants observed clients reluctant to evacuate due to past experiences, financial concerns, or mobility issues. There is room for improvement of the emergency preparedness planning routinely done with clients, as our study noted that for some clients, the needed steps to prepare were not taken. HCPs can work to identify the barriers around preparedness actions, such as why their patients do not plan to evacuate, and work with them to develop a safe plan, in conjunction with emergency response planners [7, 8]. On top of this, research is needed that critically analyzes the effectiveness of current preparedness and response planning, including how to better support those whose reported intention is to not evacuate [27, 28].

### Limitations

This study does have limitations that prevent the findings from being widely generalizable. First, this study is set in two hurricane affected areas, and included only 25 home based care providers. Second, the majority of participants were white women, and therefore this study does not represent the diverse perspective that is needed to better understand and support structurally marginalized communities. Also needed is the study of a large scope of types of disasters and communities with differing sociodemographics. However, this study is one of few that gives voice to the important insight of home based care providers during disasters and community emergencies.

## Conclusions

Home based care fulfills an essential need during a disaster, where providers continue to support their clients through all phases of a disaster to maintain minimally-interrupted care. Our study provides insights on how home based care providers and other stakeholders can address disaster-associated health challenges. We call for an emphasis on the importance of community supports, and a sustained focus on supporting structurally marginalized individuals and communities.

## Data Availability

The datasets generated and/or analyzed during this current study are not publicly available due to ongoing use of data set but are available from the corresponding author on reasonable request.

## Abbreviations

ADLs: Activities of daily living
HCPs: Home based care providers
IRB: Institutional review board
COREQ: Criteria for reporting qualitative research
FEMA: Federal Emergency Management Agency
CMS: Centers for Medicare and Medicaid Services (CMS)
LVN: Licensed Vocational Nurses
CPAP: Continuous Positive Airway Pressure
